# Nitric Oxide-Releasing Aspirin Suppresses NF-κB Signaling in Estrogen Receptor Negative Breast Cancer Cells *in Vitro* and *in Vivo*

**DOI:** 10.3390/molecules200712481

**Published:** 2015-07-09

**Authors:** Niharika Nath, Mitali Chattopadhyay, Deborah B. Rodes, Anna Nazarenko, Ravinder Kodela, Khosrow Kashfi

**Affiliations:** 1 Department of Physiology, Pharmacology and Neuroscience, Sophie Davis School of Biomedical Education, City University of New York Medical School, New York, NY 10031, USA; E-Mails: mitali@med.cuny.edu (M.C.); debrodes@optonline.net (D.B.R.); meetannanazarenko@gmail.com (A.N.); ravinder@med.cuny.edu (R.K.); 2 Department of Life Sciences, New York Institute of Technology, New York, NY 10023, USA; 3 Division of Cancer Prevention, Department of Medicine, Stony Brook University, Stony Brook, NY 11794, USA

**Keywords:** NSAIDs, NO-NSAIDs, NF-κB, oxidative stress, breast cancer, estrogen receptor

## Abstract

Estrogen receptor negative (ER(−)) breast cancer is aggressive, responds poorly to current treatments and has a poor prognosis. The NF-κB signaling pathway is implicated in ER(−) tumorigenesis. Aspirin (ASA) is chemopreventive against ER(+) but not for ER(−) breast cancers. Nitric oxide-releasing aspirin (NO-ASA) is a safer ASA where ASA is linked to an NO-releasing moiety through a spacer. *In vitro*, we investigated anti-proliferation effects of NO-ASA (*para-* and *meta-*isomers) against ER(−) breast cancer cells MDA-MB-231 and SK-BR-23, effects on NF-κB signaling, and reactive oxygen species by standard techniques. *In vivo*, effects of NO-ASA were evaluated in a mouse xenograft model using MDA-MB-231 cells. *p*-NO-ASA inhibited the growth of MDA-MB-231 and SK-BR-3 cells at 24 h, the respective IC_50_s were 13 ± 2 and 17 ± 2 μM; ASA had an IC_50_ of >3000 μM in both cell lines. The IC_50_s for *m*-NO-ASA in MDA-MB-231 and SK-BR-3 were 173 ± 15 and 185 ± 12 μM, respectively, therefore, implying *p*-NO-ASA as a stronger inhibitor of growth *p*-NO-ASA reduced cell growth by inhibiting proliferation, inducing apoptosis and causing G_0_/G_1_ cell cycle block. Activation of NF-κB was inhibited by both isomers as demonstrated by decreases in NF-κB-DNA binding and luciferase activity at 24 h, However, *m*-NO-ASA produced transient effects at 3 h such as increased NF-κB-DNA-binding, increased levels of nuclear p50, even though both isomers inhibited IκB degradation. Increase in nuclear p50 by *m*-NO-ASA was associated with translocation of p50 in to the nucleus as observed by immunoflouresence at 3 h. NO-ASA induced reactive oxygen species (ROS) as evidenced by overall increases in both H_2_DCFDA (2′,7′-dichlorodihydrofluorescein) and DHE (dihydroethidium)-derived fluorescence. Inhibition of ROS by *N*-acetyl-cysteine reversed the *m*-NO-ASA-mediated translocation of p50 in to the nucleus. In xenografts, *p*-NO-ASA inhibited tumor growth by inhibiting proliferation (PCNA and tumor volume), inducing apoptosis (TUNEL positive cells) and reducing NF-κB expression. Both isomers inhibit cancer cells, inhibit NF-κB pathway and induce ROS, and have potential as anticancer compounds.

## 1. Introduction

Breast cancer is the second leading cause of cancer deaths in women in the US [1]. It is classified as either estrogen receptor positive (ER(+)) or estrogen receptor negative (ER(−)) [2]. The latter represent 25%–30% of human breast cancers, are very aggressive and have a much poorer prognosis, given the lack of target-directed therapies. Due to the limitations of current therapies, chemoprevention emerges as a major component of breast cancer control. Selective estrogen receptor modulators such as tamoxifen and raloxifene have a well-established role in preventing breast cancer [3]. These agents, however, reduce the incidence of only ER(+) breast cancer [4] and can lead to the development of tamoxifen resistance [5], emphasizing the need to develop strategies for the chemoprevention of ER(−) breast cancer.

**Figure 1:**
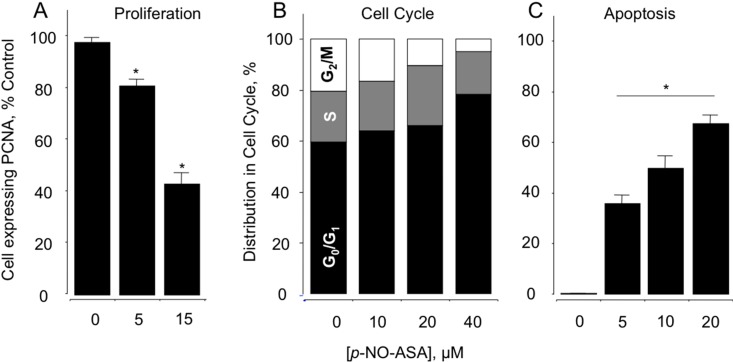
Effect of NO-ASA on MDA-MB-231 breast cancer cell kinetics. NO-ASA inhibits proliferation by altering cell cycle progression and inducing apoptosis. Cells were treated with vehicle, various concentrations of NO-ASA for 24 h and analyzed for (**A**) proliferation by PCNA antigen expression; (**B**) cell cycle phases by PI staining and flow cytometry; (**C**) cells were collected and analyzed for Annexin-V and PI labeling. The percentage of cells that were Annexin-V positive/PI negative (early apoptosis) and those that were Annexin-V positive/PI positive (late apoptosis) is shown as total apoptotic cells. In (**A**,**C**), results are mean ± SEM for three different experiments performed in duplicate, *: *p* < 0.05, compared to control. In (**B**), results are representative of two different experiments. This study was repeated twice generating results within 10% of those presented here.

Nitric oxide releasing nonsteroidal anti-inflammatory drugs (NO-NSAIDs) are a promising group of compounds for the control of cancer. They consist of a traditional NSAID and a covalently bound NO-releasing moiety (reviewed in [6,7]). The most promising of them is NO-aspirin (NO-ASA) (Figure 1). There is evidence that NO-ASA has a strong chemopreventive effect on colon and pancreatic cancer [8,9]. Furthermore, several animal and early human studies emphasize its apparent safety, at least when compared to conventional ASA [10,11].

A current approach to rational treatment development capitalizes on the knowledge of drug effects on cell signaling pathways relevant to cancer. Multiple lines of evidence implicate the NF-κB signaling pathway as playing an important role in mammary tumorigenesis. There are three sets of data which underscore the importance of NF-κB: First, activated NF-κB was detected predominantly in ER(−) *vs.* ER(+) breast tumors and mostly in ER(−) and ErbB2(+) tumors (86%) [12]. Second, activated NF-κB is associated with functional and biological significance; ER(−) breast cancer cells rely on NF-κB for aberrant cell proliferation and simultaneously avoid apoptosis [13]. Third, breast cancers that lack functional ER overexpress NF-κB-regulated genes [13]. Breast cancers often progress from a hormone-dependent, nonmetastatic, antiestrogen-sensitive phenotype to a hormone-independent, antiestrogen- and chemotherapy-resistant phenotype with highly invasive and metastatic growth properties. This progression is usually accompanied by altered function of the ER or outgrowth of ER(−) cancer cells [13]. Indeed, the chemotherapeutic resistance in ER(−) breast cancers can be accounted for by the activation of NF-κB. The clear implication of these observations is that constitutively activate NF-κB is a *bona fide* target for ER(−) breast cancer [12,14].

Previous work by us as well as others, mainly in cell lines of leukemia, colon and pancreatic cancers, indicate that these compounds could affect the NF-κB pathway [15,16,17] and that reactive oxygen species (ROS) production contributed to the suppression of NF-κB activity in leukemic cells [17]. The NO donating compound NO-ASA induced ROS, which was associated with cell cycle arrest, anti-proliferative effects and apoptosis, as demonstrated mostly in colorectal and pancreatic cell lines [18,19,20]. Among the studies in breast cancer cells with NO donating compounds, encouraging effects and possible mechanisms of NO-ASA and two other compounds, NOSH-sulindac and NOSH-naproxen, in ER(+) cells have also been demonstrated [21,22]. However, regarding the aggressive ER(−) breast cancers, mechanistic studies of NO donating ASA or its isomers in this area are lacking and interplay of NF-κB pathway with ROS, if any, have not been examined in these cells.

Regulating this pathway could prove useful for the primary or secondary prevention of ER(−) breast cancer. Therefore, we explored the effects of the *para* and *meta* isomers of NO-ASA using two ER(−) breast cancer cell lines and a xenograft model. *In vitro*, our results demonstrate that *para* and *meta* positional isomers of NO-ASA inhibit the growth of these two cell lines with the *para* isomer being more potent and that this effect is accompanied by inhibition of the NF-κB signaling and generation of ROS. The *meta* isomer of NO-ASA regulates NF-κB activity via ROS up-regulation, while the *para* isomer does not. In the xenograft model, *p*-NO-ASA reduced tumor volume, tumor mass and activated NF-κB.

## 2. Results and Discussion

### 2.1. NO-ASA Inhibits Breast Cancer Cell Growth

The effect of two NO-donating derivatives, *p-* and *m*-NO-ASA, and the parent compound, ASA were examined on cell growth. Breast cancer cell lines SK-BR-3 and MDA-MB-231, which are ER(−), were exposed to various concentrations of these compounds for 24 h and MTT assays were performed. From their growth inhibition curves, Inhibitory Concentration (IC_50_) values were calculated (Table 1). *p-*NO-ASA was very potent in inhibiting the growth of both breast cancer cell lines; the IC_50_s being 13 ± 2 and 17 ± 2 μM for MDA-MB-231 and SK-BR-3 cells, respectively. Compared to *p*-NO-ASA, *m*-NO-ASA was less potent, the corresponding IC_50_s being 173 ± 15 and 185 ± 12 μM for MDA-MB-231 and SK-BR-3 cells, respectively. In this study an IC_50_ for ASA could not be determined even though concentrations as high as 3000 μM were used. This is consistent with other studies using colon and pancreatic cancer lines where concentrations as high as 5000 μM were used without reaching an IC_50_ for cell growth inhibition after 24 h of exposure [23]. Additionally, 48 h treatments were also performed and respective IC_50_s were determined (Table 1). For 48 h intervals, IC_50_s were lower compared to that for 24 h. For example, the IC_50_ for ASA, *p*-NO-ASA, *m-*NO-ASA in MDA-MB-231 cells at 48 h were 2200 ± 180 μM, 3.3 ± 0.4 μM, 95 ± 10 μM, respectively; and in SK-BR-3 cells the corresponding values were 2550 ± 210, 5.3 ± 0.8 and 110 ± 15 μM. This implies there is a time dependent effect and biological activity in the medium of both compounds for up to 48 h. Compared with the parent ASA, the two NO-donating derivatives showed many-fold increase in potency, as determined by the ratio of the IC_50_s. For example, the ratio of IC_50_ values (ASA/NO-ASA) for *p-*NO-ASA in MDA-MB-231 and SK-BR-3 cells at 48 h were 667 and 481, indicating it to be 667-fold and 481-fold more potent than ASA. Similarly, *m-*NO-ASA was 23-fold more potent in both cell lines. For further analysis on cell growth, the triple negative cell line MDA-MB-231 was used.

**Table 1:** IC_50_ values for ASA and NO-ASA, in MDA-MB-231 and SK BR 3 breast cancer cells.

Drug	IC_50_, μM, 24 h		IC_50_, μM, 48 h	
	MDA-MB-231	SK BR-3	MDA-MB-231	SK BR-3
ASA	>3000 ^†^	>3000 ^†^	2200 ± 185	2550 ± 210
*p-*NO-ASA	13 ± 2 *	17 ± 2 *	3.3 ± 0.4	5.3 ± 0.8
*m-*NO-ASA	173 ± 15 *	185 ± 12 *	95 ± 10	110 ± 15

Cells were treated with various concentrations of ASA, *p-*NO-ASA, and *m-*NO-ASA as described under Experimental Section. Cell numbers were determined by MTT at 24 and 48 h, from which IC_50_ values were calculated. Results are mean ± S.E.M. of at least three different experiments done in duplicate. *: *p* < 0.001 compared to ASA. ^†^: Exceeded the maximum concentrations used in these studies.

### 2.2. NO-ASA Inhibits Cellular Proliferation, Alters Cell Cycle Phases and Induces Cell Death

In order to evaluate the mechanism(s) involved in the reductions of cell growth, the effect of NO-ASA was evaluated on cell renewal and cell death, two determinants of cell growth. PCNA constitute a marker of proliferation status, thus MDA-MB-231 cells were analyzed for PCNA expression after treatment with *p-*NO-ASA for 24 h. The cells showed a significant antiproliferative effect, as seen by a concentration-dependent reduction in the expression of PCNA (Figure 1A). At 5 μM the reduction was 81% ± 2% and at 15 μM it was 42% ± 5% (compared to control, *p* < 0.01). Qualitatively, similar results were obtained with *m-*NO-ASA but at higher concentrations implying lower potency (data not shown). Cell cycle transitions were examined by flow cytometry. Phases of the cell cycle were blocked by both NO-ASA positional isomers in MDA-MB-231 cells (Figure 1B). For example, following 24 h of treatment, *p-*NO-ASA induced a block of cells in the G_0_/G_1_ phase. This was evident by the increased population of cells in the G_0_/G_1_ phase that was accompanied by corresponding reductions of the proportion of cells in S and G2/M phases.

Apoptosis was measured in the cells by Annexin V-FITC/PI assay followed by flow cytometry. Cells in early apoptosis were quantified by Annexin V-positive and PI-negative cell populations. At 24 h, *m-*NO-ASA 50 and 200 μM produced approximately 56% and 66% early apoptotic cells. At higher concentrations of 300 and 500 μM, these cells likely moved into late apoptosis as the Annexin V-positive and PI-negative populations were reduced to 55% and 39%, respectively. Similarly, 5 μM *p-*NO-ASA for 24 h produced large numbers of cells, approx. 61%, in early apoptosis, while 10 and 20 μM reduced this population to 50% and 35% respectively, possibly moving them to late apoptosis. Late apoptotic populations represented by Annexin V-positivity and PI- positivity increased accordingly (Figure 1C). Therefore, *p-*NO-ASA contributes its effects on cell growth by a combination of effects on proliferation, cell cycle arrest and apoptosis. It has been reported by us earlier through several studies that both isomers of NO-ASA also acts on these major cellular processes in various cell types. This agrees well with our current understanding that these effects of NO-ASA are independent of cell type [23].

### 2.3. NO-ASA Inhibits NF-κB Signaling Pathway

**Figure 2:**
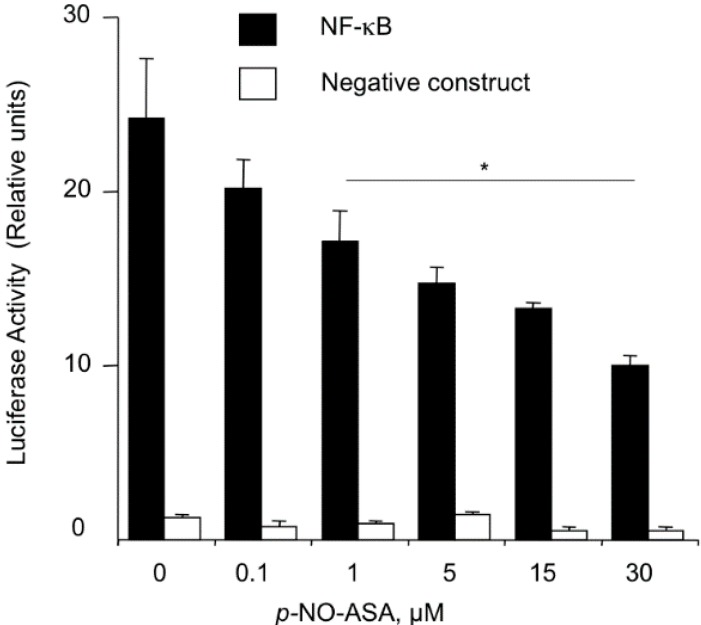
NO-ASA suppresses the NF-κB signal transduction pathway. MDA-MB-231 breast cancer cells were transiently transfected with either the pNF-κB-Luc construct or with the control reporter plasmid, which lacks the κB binding sequences, as described in Experimental Section. After overnight incubation, transfected cells were treated with *p*-NO-ASA for 18 h. *p*-NO-ASA suppressed this signaling pathway with an IC_50_ of 17 ± 2 μM. Results are mean ± SEM for three different experiments, *: *p* < 0.05.

*p-NO-ASA inhibits NF-*κ*B-Luc reporter activity:* Activation of the transcription factor NF-κB involves its translocation into the nucleus, where it binds to the appropriate DNA regulatory sequences. Normally, the DNA transportation domain of NF-κB is bound by IκB, thereby, sequestering the heterodimer in the cytoplasm. Hence, activation of NF-κB is regulated by the ubiqitination of IκB. NF-κB is constitutively expressed in most cancer cell lines and plays a major role in cell survival, specifically, proliferation and anti-apoptosis. First, we examined if NF-κB signaling is altered by *p-*NO-ASA. MDA-MB-231 cells were transfected with 4× κB-Luc reporter plasmid or the negative construct lacking the κB binding sequences. Post overnight incubation, transfected cells were treated with *p*-NO-ASA 0.1 to 30 κM for 18 h. *p-*NO-ASA reduced luciferase activity in a concentration dependent manner (Figure 2), and IC_50_ for this reduced transcriptional activity was 17 μM.

### 2.4. NO-ASA Inhibits NF-κB DNA-Binding Activity

**Figure 3:**
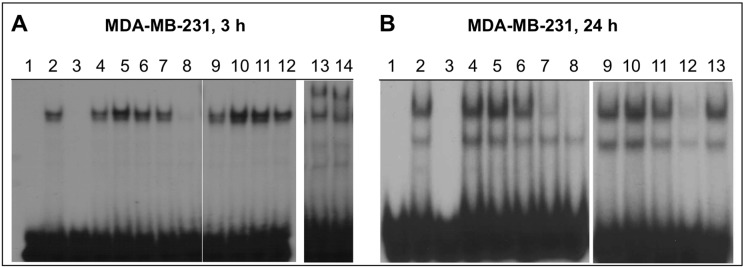
NO-ASA inhibits NF-κB-DNA binding. For this electrophoretic mobility shift assay (EMSA) of NF-κB activation, MDA-MB-231 cells were treated for 3 or 24 h with *p-*NO-ASA or *m-*NO-ASA or ASA. Panel **A**: MDA-MB-231 cells, treated for 3 h as follows. Lanes; 1: No extract; 2: control; 3: control + specific competitor; 4: control + nonspecific competitor; 5–8: *p-*NO-ASA, 1, 5, 10, and 20 μM, respectively; 9–11: *m-*NO-ASA, 50, 200, and 400 μM, respectively; 12: ASA, 1 mM; 13 and 14: supershift assay using p50 and p65 antibodies, respectively. Panel **B**: MDA-MB-231 cells, treated for 24 h as follows. Lanes; 1: No extract; 2: untreated extract; 3: control + specific competitor; 4: control + nonspecific competitor; 5–8: *p-*NO-ASA, 1, 5, 10, and 20 μM, respectively; 9: control; 10–12: *m-*NO-ASA, 50, 200, and 400 μM, respectively; 13: ASA, 1 mM.

We determined whether NO-ASA affects the NF-κB-DNA interaction in MDA-MB-231 cells by gel shift assays on nuclear extracts. Cells were treated for 3 or 24 h with *p-* or *m-*NO-ASA. Nuclear extracts were examined for binding to a P^32^-labeled 22-oligomer (probe) bearing the NF-κB binding sites. As shown in Figure 3, DNA-protein complexes representing NF-κB-bound probe were obtained with untreated extracts (Panel A, control, lane 2). Competition with the unlabeled probe as specific inhibitor caused ablation of the band (lane 3) while supershift with p65 and p50 antibodies confirmed that the NF-κB in the extract is the p65/p50 form (lanes 13 and 14). *p*-NO-ASA decreased the basal level of NF-κB bound DNA in a concentration- and a time-dependent manner. At 3 h, *p*-NO-ASA 20 μM strongly decreased DNA binding (Panel A, lane 8), while at 24 h, 10 μM and 20 μM were both effective (Panel B, lanes 7 and 8). In contrast, *m*-NO-ASA produced a biphasic effect; at 3 h there was increased DNA-binding at 200 and 400 μM (Panel A, lanes 10, 11) while at 24 h there was a sharp decrease at these concentrations (Panel B, lanes 11, 12). Finally, traditional ASA 1000 μM had no significant effect at any time point (Panel A, lane 12 and Panel B, lane 13). Therefore, we conclude that effects of NO-ASA involve both p50 and p65 subunits of NF-κB in MDA-MB-231 cells, and that reduced DNA binding results in reduced activation as observed in the reporter assay.

### 2.5. Effect of NO-ASA on Protein Levels of NF-κB Subunit p50

**Figure 4:**
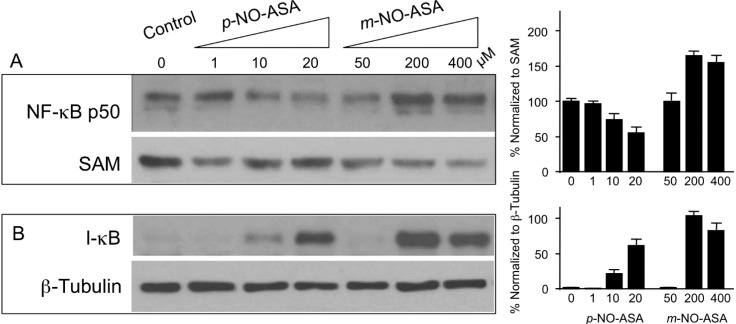
NO-ASA affects NF-κB p50 and IκB levels. MDA-MB-231 cells were treated with indicated concentrations *p-* or *m*-NO-ASA. Immunodetection of protein levels was performed on (**A**) nuclear extracts for NF-κB p50 levels using SAM68 as internal control for nuclear proteins; and (**B**) cytoplasmic extracts for IκB levels, and β-Tubulin as internal loading control. Results are representative of two different experiments; bar graphs represents mean ± range of the two experiments.

Nuclear extracts were examined for protein levels of p50 subunit of NF-κB in order to determine if translocation to nucleus is altered leading to reduced DNA-binding. Cells were treated with *p*-NO-ASA for 3 h, and protein extracts of nucleus and cytoplasm were obtained. Immunoblotting and normalization of p50 with nuclear Sam68 as internal control revealed reduced levels of p50 subunit in response to *p*-NO-ASA (Figure 4A and bar graphs). Therefore, *p*-NO-ASA inhibits nuclear translocation of p50, which agrees with the reporter assay results. It is well established that the inhibitory molecule IκBα binds to the NF-κB p50 and p65 subunits in the cytoplasm. Proteolytic degradation of this inhibitory subunit allows dissociation of binding and translocation of NF-κB subunits into the nucleus. We examined if reduced translocation of p50 into the nucleus by *p*-NO-ASA occurs by suppression of IκBα degradation. Cytoplasmic extracts showed *p*-NO-ASA increased amounts of IκBα implying inhibition of its degradation (Figure 4B and bar graphs). Therefore, *p*-NO-ASA inhibits IκBα degradation, leading to its continued binding to p50, and inhibiting p50 translocation to the nucleus. In contrast, *m*-NO-ASA also inhibits IκBα degradation, however it does not prevent translocation of p50 in the nucleus, rather it promotes translocation to the nucleus, which explains clearly the increased DNA-binding observed at 3 h (Figure 4A). At this time it is not yet clear how this increase in p50 occurs in the nucleus based on current understanding of NF-κB regulation. Other effects of *m*-NO-ASA on nuclear transport and/or nuclear pores may also need to be examined as a separate course of study to shed more light on this observation. It is well known that NF-κB regulates cell proliferation and cell death [24]. Our previous work has demonstrated that NO-ASA inhibits cell growth through its combined effects on cell proliferation and apoptosis. The IC_50_ for cell growth inhibition and the IC_50_ for inhibition of NF-κB-DNA binding correlate well and imply a role of NF-κB in the dynamics of growth inhibition. The effects of NO-ASA on NF-κB pathway may be an important sole contributor. NF-κB is activated preferentially in many human breast cancers, for example constitutive NF-κB activation is shown to be preferentially involved in proliferation of basal-like subtype breast cancer cells [25].

### 2.6. NO-ASA Induces ROS

**Figure 5:**
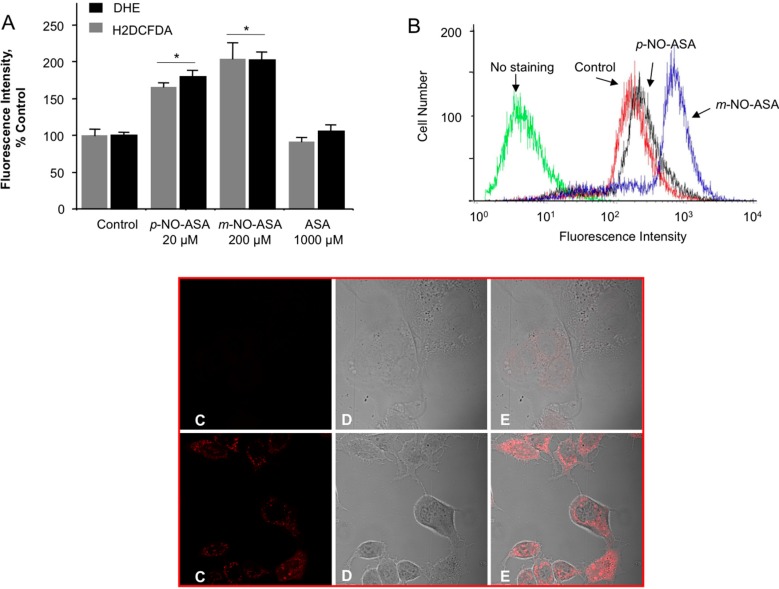
NO-ASA induces ROS levels. MDA-MB-231 cells were treated with NO-ASA for 1 h followed by staining with a general ROS probe DCFDA or DHE, which detects superoxide anions (Panel A). (**A**) Representative histogram for DCFDA is shown in (**B**). Values are the mean ± SEM of three independent experiments. *: *p* < 0.05 compared to untreated controls. NO-ASA also produces ROS in mitochondrion. MDA-MB-231 cells were treated with 200 μM *m-*NO-ASA for 3 h. Upper panel C represents vehicle-treated and the lower panel shows drug-treated cells. The red signal in panel C is MitoSox, which depict mitochondrial ROS. Panel D are unstained images. Panel E shows overlaid images from C and D.

We examined whether NO-ASA induced ROS levels. H2DCFDA is a molecular probe that detects more than ten individual reactive species including peroxides [26,27]. DHE is a selective probe for superoxide anion (O_2_^•−^) [28]. Cells were treated with indicated concentrations of *para* and *meta* NO-ASA based on their IC_50_ values for growth inhibition for 1 h and analyzed for levels of intracellular peroxides as described in Experimental Section. Compared with control, 20 μM *p-*NO-ASA and 200 μM *m-*NO-ASA increased the population of cells representing DHE- and H2DCFDA-dependent fluorescence, indicating an induction of intracellular peroxides and superoxide anion O_2_^•−^, respectively (Figure 5A,B). ROS generation by IC_50_ levels of NO-ASA was almost twofold compared to the control cells and the *para* isomer produced less ROS than the *meta* isomer.

We further performed microscopic evaluation in live cells to directly detect mitochondria-derived O_2_^•−^ using MitoSOX Red. This study was performed with *m-*NO-ASA. MDA-MB-231 cells were treated with 200 μM *m-*NO-ASA for 3 h. Compared with untreated control, *m*-NO-ASA produced strong staining for ROS (or mitochondrial superoxide) in mitochondrion as visualized by MitoSox staining in live cells (Figure 5, Panels C–E). This indirectly implies that mitochondrial superoxide dismutase (SOD) responds to *m*-NO-ASA. As ROS induction maybe a compartment specific due to presence of different types of SODs in cytoplasm and mitochondria [29] this opens up avenues for dissecting the pathways and enzymes involved.

ROS are important signaling molecules [30]. Increase in oxidative stress appears to be an early event as ROS levels are induced at 1 h. The oxidative stress produced by ROS maybe a mechanism related to apoptosis induction in these cells, in particular because we found comparatively stronger apoptosis by both compounds as opposed to strong cell cycle alterations. However, *in vivo* studies showed NO-ASA spares the GI from the toxicity of the parent compound ASA [31,32]. Therefore, the fact that NO-ASA is GI sparing *in vivo* and yet induces ROS *in vitro* implies that the events are not mutually exclusive. This underscores the need for caution in translating *in vitro* data to *in vivo* observations. Similarly, NO-ASA induced the expression of COX-2 in various cell lines, whereas in animals it inhibited COX activity and expression of prostaglandins [15,32].

**Figure 6:**
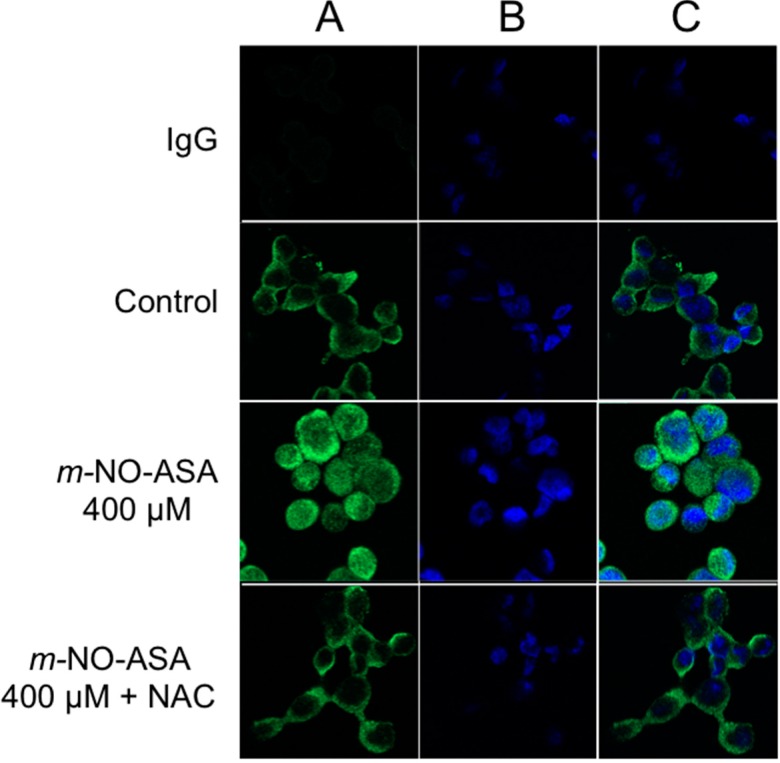
ROS is necessary for p50 translocation to nucleus by *m*-NO-ASA. MDA-MB-231 cells were treated with *m*-NO-ASA for 3 h, immunofluorescence of p50 was performed using anti-p50 antibody or IgG control and a Two-Photon Laser Scanning Confocal Microscope. DAPI was used to stain the nucleus. Panel **A**, top to bottom. *m*-NO-ASA increased p50 levels in the nucleus compared to control, which was reduced substantially by NAC. Panel **B**, DAPI stained nucleus; and Panel **C**, merged.

Regarding NF-κB, very high ROS levels can push cells to apoptosis and overall production of huge oxidative stress and cell death has been reported associated with inhibition of the NF-κB signaling pathway [33]. Further, ROS can cause redox modifications that inhibit activation of NF-κB [34], therefore, NF-κB signaling and ROS may have reportedly associated roles in cell death mediated by NO-ASA. We investigated the relationship between increases in ROS and NO-ASA-mediated modulation of NF-κB in these cells and determined whether ROS is necessary for *m*-NO-ASA-mediated increases in nuclear NF-κB p50. For this, we inhibited ROS by a common ROS scavenger, *N*-acetyl cysteine (NAC). Cells were pretreated with NAC 10 mM followed by *m*-NO-ASA for 3 h, and immunofluorescence for p50 was performed. *m*-NO-ASA (400 μM) increased p50 levels in the nucleus compared to control, as shown by an increase in signals in the nucleus (Figure 6 Panel A,) which was reduced substantially by NAC. In contrast, *p*-NO-ASA showed no detectable changes in p50 immunofluorescence at any concentration or with NAC compared to untreated control (data not shown) possibly because basal levels were low for detection by this method. Therefore, ROS is necessary for *m*-NO-ASA-mediated translocation of NF-κB p50 into the nucleus. Considering that more ROS was produced by *m-*NO-ASA than *p-*NO-ASA (Figure 5), it appears that ROS has a stronger role in modulating p50 by *m*-NO-ASA, and that overall cell growth inhibition by *m*-NO-ASA and *p-*NO-ASA may be attributed to differences in mechanisms. While generation of ROS is generally associated with higher NF-κB activity [35], inhibitory effect on NF-κB activation in lung epithelial cells is also known [36].

### 2.7. NO-ASA Modulates Cell Proliferation and Inhibits NF-κB Activation in a ER(−) Xenograft Model

**Figure 7:**
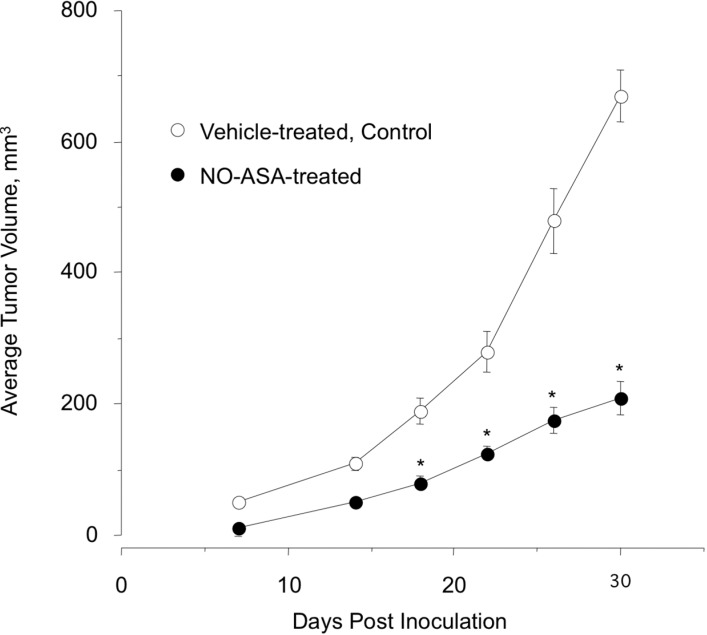
Female athymic nude mice were divided into two groups (*n* = 10 per group). One group was gavaged daily with *p-*NO-ASA (100 mg/kg body weight) suspended in methylcellulose and the other group was gavaged with the vehicle. After one week of treatment, animals in both groups were implanted s.c. in the right flank with MDA-MB-231 cells (3 × 10^6^) suspended in 50% Matrigel. In the vehicle-treated animals, small tumors were palpable after one week. Tumor size was measured every four days, *: *p* < 0.05.

To explore the relevance of our *in vitro* findings to breast cancer, we used a tumor xenograft model. In this prevention model, female athymic nude mice were pre-initiated by daily administration of *p-*NO-ASA (100 mg/kg, gavage) and a control group was gavaged with the vehicle for seven days, followed by subcutaneous implantation of MDA-MB-231 cells (3 × 10^6^) in the right flank. Daily administration of the test agents continued to the end of the experiment. At the end of the study, *p-*NO-ASA-treated mice showed a considerable reduction in tumor volume compared with the control group (Figure 7). The mean tumor volumes at sacrifice for the control group and *p-*NO-ASA were 670 ± 40 mm^3^ and 210 ± 8 mm^3^, respectively. This is equivalent to a mean reduction of 68% (*p* < 0.05).

**Figure 8:**
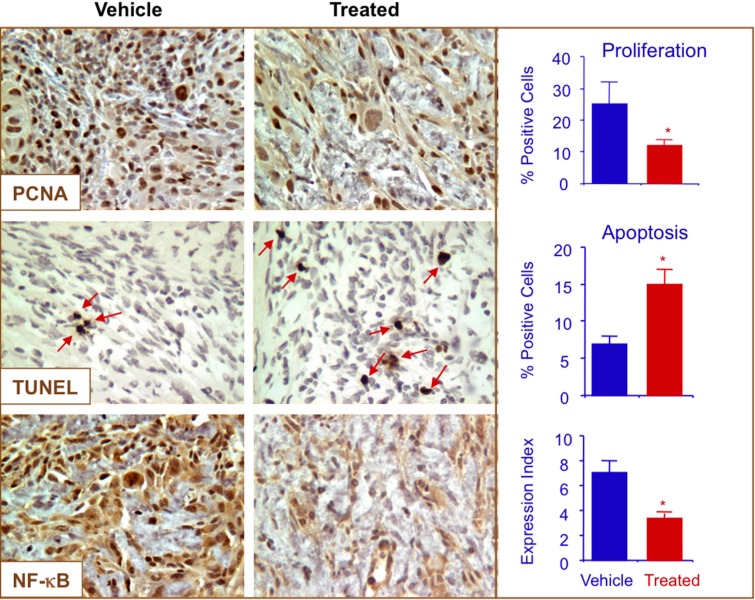
*p*-NO-ASA inhibits proliferation, induces apoptosis and decreases NF-κB p65 *in vivo*. Tumors were sectioned, probed, and scored as described in the Experimental Section. Average mitotic index at sacrifice was determined by PCNA, TUNEL and p65 staining (*: *p* < 0.05). Red arrows indicate apoptotic cells. Representative fields used for quantification are shown.

Immunohistochemical staining of the tumors were performed for expression of NF-κB p65, PCNA and for apoptotic cells by TUNEL staining. Compared to control tumors, the treated group showed a strong reduction in expression of PCNA (from 26% ± 9% to 12% ± 2%) and increase in apoptotic cells (from 6% ± 2% to 15% ± 3%), respectively. NF-κB expression was also significantly reduced by *p-*NO-ASA (Figure 8). Therefore, *p*-NO-ASA suppressed tumor growth by a combination of increased apoptosis and reduced cell proliferation.

## 3. Experimental Section

### 3.1. Reagents

The two positional isomers of NO-ASA, *para* isomer [2-(acetyloxy)benzoic acid 4-(nitrooxy methyl)phenyl ester]]; and the *meta* isomer [2-(acetyloxy)benzoic acid 3-(nitrooxy methyl)phenyl ester], were synthesized according to published methods [37]. ASA was obtained from Sigma (St Louis, MO, USA). Stock solutions (100 mM) were made in DMSO; final DMSO concentration was adjusted in all media to 1%.

### 3.2. Breast Cancer Cells

ER(−) breast cancer cell lines of two different subtypes were used in this study, MDA-MB-231 (ER−, PR−, HER2−, *i.e.*, triple negative, basal-subtype, highly metastatic) and SK-BR-3 (ER−, PR−, HER2+ *i.e.*, Her2 overexpression-subtype) were obtained from American Type Culture Collection (ATCC, Manassas, VA, USA). All cells were grown per ATCC recommended instructions.

### 3.3. Cell Growth Inhibition Assay

The growth inhibitory effect of NO-ASA on breast cancer cells was measured using a colorimetric MTT assay kit (Roche). Briefly, cells were plated in 96-well plates at a density of 13.7 × 10^3^ cells/well and, following overnight incubation, *p-* or *m-*NO-ASA, or ASA, was added to the culture medium. At indicated time periods of treatment, viable cells were quantified with MTT substrate according to the manufacturer’s instructions. Growth inhibition is expressed as percentage of the corresponding control.

### 3.4. Cell Proliferation

Proliferating cell nuclear antigen (PCNA) was determined using an ELISA Kit (Calbiochem, La Jolla, CA, USA), in accordance with the manufacturers protocol and read on spectrophotometric plate reader.

### 3.5. Flow Cytometry for Phase Distribution in the Cell Cycle and Detection of Apoptosis

*Cell Proliferation and Cell Cycle Phases*: Cell Proliferation and Cell Cycle Analysis were performed as previously described [38]. Cell cycle phase distributions were obtained using a Coulter Profile XL equipped with a single argon ion laser. For each subset, we analyzed >10,000 events. All parameters were collected in listmode files. Data were analyzed on an XL Elite Work station (Coulter) using the Software programs Multigraph™ and Multicycle™. The percentage of cells in G_0_/G_1_, G_2_/M, and S phases was determined form DNA content histograms.

### 3.6. Apoptosis Assay

Cell death was FITC-labeled Annexin V and propidium iodide (PI), using an Annexin V-FITC apoptosis detection kit, (BD Biosciences, San Jose, CA, USA), according to the manufacturer’s instructions. Treated cells (floating and adherent cells) were collected, resuspended in an Annexin V binding buffer and transferred to test tubes containing FITC-labeled Annexin V and PI. The cells were then incubated for 15 min at room temperature in dark, and analyzed by flow cytometry. Annexin V-FITC-positive/PI-negative cells were regarded as a measure of early apoptosis. In addition, qualitative determination of apoptosis was determined by fluorescence microscopy of cells stained with 4,6-diamidino-2-phenylindole (DAPI; Accurate Chemicals, Westbury, NY, USA). For each sample, at least five fields were examined. The morphological criteria used to identify apoptosis included cytoplasmic and nuclear shrinkage, chromatin condensation, and membrane blebbing.

### 3.7. Cell Transfection and NF-κB Reporter Assays

MDA-MB-231 breast cancer cells were transiently transfected with 800 ng of either the pNF-κB-Luc construct, which has 4× binding sites or the with the control reporter plasmid pTA-Luc construct representing the negative control (lacking the κB binding sequences) using Lipofectamine 2000 (Invitrogen, Carlsbad, CA, USA) transfection reagent. Cells were co-transfected with 400 ng β-gal construct to control for transfection efficiency. After overnight incubation, transfected cells were treated with NO-ASA for 18 h. Lysates were examined for luciferase activity (Promega, Madison, WI, USA) and normalized with β-galactosidase activity for transfection efficiency.

### 3.8. Electrophoretic Mobility Shift Assays (EMSA)

Briefly, cells were incubated with aspirin, *p-* or *m-*NO-Aspirin or solvent (DMSO) for the indicated times and concentrations. Nuclear extracts were prepared following standard protocols [39] and resuspension of nuclear pellet in extraction buffer. Proteins obtained in the cellular lysis step were collected, stored and used in experiments requiring cytoplasmic lysates. Nuclear extracts were subjected to EMSA to evaluate NF-κB activation. The NF-κB DNA binding activity was assessed by reacting 20 μg of nuclear extract from control and treated cells with 33 nmol of a ^32^P-end-labeled 22-mer double-stranded NF-κB oligonucleotide (AGTTGAGGGGAC TTTCCCAGGC; Promega). The specificity of binding was tested by competition reactions, using a 50-fold excess of unlabeled NF-κB (specific) or AP-1 (nonspecific) consensus oligonucleotides. Supershift analysis was employed where extracts were incubated with antibodies against the NF-κB subunits p50 and p65 prior to the addition of probe. The complexes were separated on a 5% gel following standard protocols.

### 3.9. Western Blotting

Nuclear and cytoplasmic extracts as prepared as prepared above were examined for protein levels after they were fractionated by sodium dodecyl sulfate–polyacrylamide gel electrophoresis and transferred onto nitrocellulose membranes. Immunodetection using antibodies against the NF-κBp50, IκBalpha, Sam68 or β-tubulin (Santa Cruz Biotechnology, Santa Cruz, CA, USA) was done for 1 h at room temperature or overnight at 4 °C. The membranes were developed by the enhanced chemiluminescence system (Amersham Biosciences, Piscataway, NJ, USA).

### 3.10. Determination of Reactive Oxygen Species (ROS)

MDA-MB 231 cells (0.3 × 10^6^ cells/well) were plated in 6-well plates for 24 h after which they were treated with various concentrations of *p-*NO-ASA for 1 h. Cells were then trypsinized, washed once in PBS resuspended and then incubated for 30 min at 37 °C in the dark with the oxidation-sensitive fluorescent probes 2′,7′-dichlorodihydrofluorescein diacetate (H2DCFDA, 10 μM) or dihydroethidium (DHE, 5 μM) (Molecular Probes, Life Technologies, NY, USA). Fluorescence intensity was then measured by flow cytometry using a FACS Calibur (BD Bioscience, San Jose, CA, USA). H2DCFDA is a probe for H_2_O_2_, peroxyl radical, including both alkylperoxyl and hydroperoxyl, while DHE is an intracellular probe that preferentially measures superoxide anion [40,41]. A minimum of 10,000 events were analyzed and expressed as fluorescent intensity *vs.* events. We also used the fluoroprobe MitoSOX Red which has a high sensitivity for superoxide detection within the mitochondria compared with the cytosol and therefore is used to directly and selectively to detect superoxide in the mitochondria of live cells by flourescence microscopy [41].

For immunofluorescence, cells were washed with phosphate-buffered saline and fixed with methanol at 20 °C for 10 min. Nuclei were premeabilized by treatment with 0.1% Triton X100/phosphate-buffered saline for 5 min. Cells were blocked in 2% BSA/phosphate-buffered saline for 15 min and incubated with rabbit p65, p50 or IgG control antibody (Santa Cruz/1:200) for 1 h at room temperature. Subsequently, cells were incubated with Alexa Fluor^®^ 488 anti-rabbit secondary antibody for 1 h at 4 °C (1:2000). The Zeiss LSM 510 META NLO Two-Photon Laser Scanning Confocal Microscope System was used for detection of staining.

### 3.11. Xenografts

Female athymic nude mice, 8-weeks-old, were divided into 2 groups (*n* = 10 per group). One group was gavaged daily with *p-*NO-ASA (100 mg/kg body weight) suspended in methylcellulose and the other group was gavaged with the vehicle. After one week of treatment, animals in both groups were implanted s.c. in the right flank with MDA-MB-231 cells (3 × 10^6^) suspended in 50% Matrigel (BD Biosciences, San Jose, CA, USA). Both groups had free access to control diet AIN-76A (Research Diets, Inc., New Brunswick, NJ, USA) and water. Mice were evaluated daily for signs of toxicity and were weighed weekly. In the untreated animals, small tumors were palpable after one week. Tumor size was measured every 4 days using electronic calipers, the tumor volumes were calculated using the following formula: length × width^2^/2. Thirty days post inoculation, the mice were sacrificed, the tumors excised and fixed in 10% buffered formalin for immunohistochemical studies. The animal protocol was approved by the Institutional Animal Care and Use Committee at Stony Brook University.

### 3.12. Immunohistochemistry

Paraffin embedded sections were dewaxed, rehydrated and microwave heated for 15 min in 0.01 M citrate acid buffer (pH 6.0) for antigen retrieval. Then, 3% hydrogen peroxide was applied to block endogenous peroxidase activity for 15 min and normal horse serum as blocking solution was incubated for 15 min. The primary antibody or isotype IgG control was applied and incubated overnight at 4 °C. Slides were washed 3 times with PBS, each for 5 min. The biotinylated secondary antibody and the streptavidin-biotin complex (Zymed Laboratories, South San Francisco, CA, USA) were applied, each for 30 min at room temperature with an interval washing. After rinsing with PBS, the slides were immersed for 5 min in the substrate 3,3′-diaminobenzidine (DAB, Sigma) 0.4 mg/mL with 0.003% hydrogen peroxide, then rinsed with distilled water, counterstained with hematoxylin, dehydrated and cover-slipped. Antibodies: PCNA 1:100, (Santa Cruz Biotechnology, Santa Cruz, CA, USA); NF-κB p65 (which recognizes activated NF-κB) 1:100, was from Chemicon International (Temecula, CA, USA).

### 3.13. TUNEL Staining

TUNEL staining was performed using the In Situ Cell Death Detection kit (Roche Applied Science, Indianapolis, IN, USA) following the manufacturer’s instructions. Briefly, 4 μm thick formalin-fixed, paraffin-embedded tissue sections were deparaffinized and rehydrated. Endogenous peroxidase activity was quenched by hydrogen peroxide and tissue protein was hydrolyzed with proteinase K (Roche Applied Science, Indianapolis, IN, USA). Negative control: sections incubated with Label Solution (without TdT enzyme). All other sections were incubated with TUNEL reaction mixture (fluorescein-labeled nucleotides) at 37 °C for 1 h in a humid chamber, incubated with Converter-POD solution (anti-fluorescein antibody conjugated with POD) for 30 min at 37 °C, treated with DAB and counterstained with hematoxylin.

### 3.14. Scoring the Expression of Biomarkers

For each animal and treatment group, 5 slides were prepared and scored by a pathologist blind to the identity of the specimens (400×). For PCNA, and TUNEL staining, cells with a brown nucleus were considered labeled and those with a blue nucleus unlabeled. For each, the percentage of positive cells over the total cells counted was calculated. For NF-κB the following semi-quantitative scoring system was used. The *extent of staining* was graded as follows: 0 = no staining; 1+ = ≤25% of cells positive; 2+ = 26%–50% of cells positive; and 3+ = ≥51% of cells positive. The *intensity of staining* was scored as follows: 0 = no staining; 1+ = faint; 2+ = moderate; 3+ = strong. 1+, 2+ and 3+ were recorded as 1, 2 and 3 points, respectively. To compare differences in staining, an Expression index (EI) was calculated by the following formula: EI = Extent of staining × Intensity of staining, as described previously [9].

### 3.15. Statistics

*In vitro* data are presented as means ± SEM for at least 3 different sets of plates and treatment groups. Xenograft data, tumor volume and mass are presented as means ± SEM for 10 animals in each group. Statistical comparison among the groups was performed using Student’s *t-*test.

## 4. Conclusions

Overall, these studies demonstrate that both isomers of NO-ASA have growth inhibitory effects that are associated with inhibition of the NF-κB pathway. NF-κB is known to be redox-sensitive and ROS production is likely to be a component of its regulation. A combination of these mechanisms has demonstrated potential anti-cancer activity in ER(−) breast cancer. *In vivo* effects have demonstrated strong benefit against ER(−) xenografts.

## References

[1] DeSantis C., Ma J., Bryan L., Jemal A. (2014). Breast cancer statistics, 2013. CA A Cancer J. Clin..

[2] Schnitt S.J., Guidi A.J. (2009). Diseases of the Breast.

[3] Reeder J.G., Vogel V.G. (2007). Breast cancer risk management. Clin. Breast Cancer.

[4] Brown P.H., Lippman S.M. (2000). Chemoprevention of breast cancer. Breast Cancer Res. Treat..

[5] Schiff R., Massarweh S., Shou J., Osborne C.K. (2003). Breast cancer endocrine resistance: How growth factor signaling and estrogen receptor coregulators modulate response. Clin. Cancer Res..

[6] Kaza C.S., Kashfi K., Rigas B. (2002). Colon cancer prevention with NO-releasing NSAIDs. Prostaglandins Other Lipid Mediat..

[7] Rigas B., Kashfi K. (2004). Nitric-oxide-donating NSAIDs as agents for cancer prevention. Trends Mol. Med..

[8] Williams J.L., Kashfi K., Ouyang N., del Soldato P., Kopelovich L., Rigas B. (2004). NO-donating aspirin inhibits intestinal carcinogenesis in Min (APC(Min/+)) mice. Biochem. Biophys. Res. Commun..

[9] Ouyang N., Williams J.L., Tsioulias G.J., Gao J., Iatropoulos M.J., Kopelovich L., Kashfi K., Rigas B. (2006). Nitric oxide-donating aspirin prevents pancreatic cancer in a hamster tumor model. Cancer Res..

[10] Wallace J.L., Reuter B., Cicala C., McKnight W., Grisham M.B., Cirino G. (1994). Novel nonsteroidal anti-inflammatory drug derivatives with markedly reduced ulcerogenic properties in the rat. Gastroenterology.

[11] Fiorucci S., Santucci L., Gresele P., Faccino R.M., del Soldato P., Morelli A. (2003). Gastrointestinal safety of NO-aspirin (NCX-4016) in healthy human volunteers: A proof of concept endoscopic study. Gastroenterology.

[12] Biswas D.K., Shi Q., Baily S., Strickland I., Ghosh S., Pardee A.B., Iglehart J.D. (2004). NF-kappa B activation in human breast cancer specimens and its role in cell proliferation and apoptosis. Proc. Natl. Acad. Sci. USA.

[13] Nakshatri H., Bhat-Nakshatri P., Martin D.A., Goulet R.J., Sledge G.W. (1997). Constitutive activation of NF-kappaB during progression of breast cancer to hormone-independent growth. Mol. Cell. Biol..

[14] Biswas D.K., Dai S.C., Cruz A., Weiser B., Graner E., Pardee A.B. (2001). The nuclear factor kappa B (NF-kappa B): A potential therapeutic target for estrogen receptor negative breast cancers. Proc. Natl. Acad. Sci. USA.

[15] Williams J.L., Nath N., Chen J., Hundley T.R., Gao J., Kopelovich L., Kashfi K., Rigas B. (2003). Growth inhibition of human colon cancer cells by nitric oxide (NO)-donating aspirin is associated with cyclooxygenase-2 induction and beta-catenin/T-cell factor signaling, nuclear factor-kappaB, and NO synthase 2 inhibition: Implications for chemoprevention. Cancer Res..

[16] Williams J.L., Ji P., Ouyang N., Liu X., Rigas B. (2008). NO-donating aspirin inhibits the activation of NF-kappaB in human cancer cell lines and Min mice. Carcinogenesis.

[17] Khan N.I., Cisterne A., Baraz R., Bradstock K.F., Bendall L.J. (2012). *para*-NO-Aspirin inhibits NF-kappaB and induces apoptosis in B-cell progenitor acute lymphoblastic leukemia. Exp. Hematol..

[18] Gao L., Williams J.L. (2012). Nitric oxide-donating aspirin induces G2/M phase cell cycle arrest in human cancer cells by regulating phase transition proteins. Int. J. Oncol..

[19] Zhou H., Huang L., Sun Y., Rigas B. (2009). Nitric oxide-donating aspirin inhibits the growth of pancreatic cancer cells through redox-dependent signaling. Cancer Lett..

[20] Sun Y., Chen J., Rigas B. (2009). Chemopreventive agents induce oxidative stress in cancer cells leading to COX-2 overexpression and COX-2-independent cell death. Carcinogenesis.

[21] Nath N., Vassell R., Chattopadhyay M., Kogan M., Kashfi K. (2009). Nitro-aspirin inhibits MCF-7 breast cancer cell growth: Effects on COX-2 expression and Wnt/beta-catenin/TCF-4 signaling. Biochem. Pharmacol..

[22] Kodela R., Chattopadhyay M., Kashfi K. (2013). Synthesis and biological activity of NOSH-naproxen (AVT-219) and NOSH-sulindac (AVT-18A) as potent anti-inflammatory agents with chemotherapeutic potential. Med. Chem. Commun..

[23] Kashfi K., Ryan Y., Qiao L.L., Williams J.L., Chen J., del Soldato P., Traganos F., Rigas B. (2002). Nitric oxide-donating nonsteroidal anti-inflammatory drugs inhibit the growth of various cultured human cancer cells: Evidence of a tissue type-independent effect. J. Pharmacol. Exp. Ther..

[24] Karin M. (2006). Nuclear factor-kappaB in cancer development and progression. Nature.

[25] Yamaguchi N., Ito T., Azuma S., Ito E., Honma R., Yanagisawa Y., Nishikawa A., Kawamura M., Imai J., Watanabe S. (2009). Constitutive activation of nuclear factor-kappaB is preferentially involved in the proliferation of basal-like subtype breast cancer cell lines. Cancer Sci..

[26] Bass D.A., Parce J.W., Dechatelet L.R., Szejda P., Seeds M.C., Thomas M. (1983). Flow cytometric studies of oxidative product formation by neutrophils: A graded response to membrane stimulation. J. Immunol..

[27] LeBel C.P., Ischiropoulos H., Bondy S.C. (1992). Evaluation of the probe 2′,7′-dichlorofluorescin as an indicator of reactive oxygen species formation and oxidative stress. Chem. Res. Toxicol..

[28] Becker L.B., vanden Hoek T.L., Shao Z.H., Li C.Q., Schumacker P.T. (1999). Generation of superoxide in cardiomyocytes during ischemia before reperfusion. Am. J. Physiol..

[29] Buettner G.R. (2011). Superoxide dismutase in redox biology: The roles of superoxide and hydrogen peroxide. Anti-Cancer Agents Med. Chem..

[30] Jhorar R., Sharma R., Kaur A., Mukherjee T.K. (2015). Role of reactive oxygen species in estrogen dependent breast cancer complication. Anti-Cancer Agents Med. Chem..

[31] Rao C.V., Reddy B.S., Steele V.E., Wang C.X., Liu X., Ouyang N., Patlolla J.M., Simi B., Kopelovich L., Rigas B. (2006). Nitric oxide-releasing aspirin and indomethacin are potent inhibitors against colon cancer in azoxymethane-treated rats: Effects on molecular targets. Mol. Cancer Ther..

[32] Rao C.V., Joseph S., Gao L., Patlolla J.M., Choi C.I., Kopelovich L., Steele V.E., Rigas B. (2008). Pharmacokinetic and pharmacodynamic study of NO-donating aspirin in F344 rats. Int. J. Oncol..

[33] Kiessling M.K., Klemke C.D., Kaminski M.M., Galani I.E., Krammer P.H., Gulow K. (2009). Inhibition of constitutively activated nuclear factor-kappaB induces reactive oxygen species- and iron-dependent cell death in cutaneous T-cell lymphoma. Cancer Res..

[34] Han D., Ybanez M.D., Ahmadi S., Yeh K., Kaplowitz N. (2009). Redox regulation of tumor necrosis factor signaling. Antioxid. Redox Signal..

[35] Schreck R., Rieber P., Baeuerle P.A. (1991). Reactive oxygen intermediates as apparently widely used messengers in the activation of the NF-kappa B transcription factor and HIV-1. EMBO J..

[36] Moodie F.M., Marwick J.A., Anderson C.S., Szulakowski P., Biswas S.K., Bauter M.R., Kilty I., Rahman I. (2004). Oxidative stress and cigarette smoke alter chromatin remodeling but differentially regulate NF-kappaB activation and proinflammatory cytokine release in alveolar epithelial cells. FASEB J..

[37] Penning T.D., Talley J.J., Bertenshaw S.R., Carter J.S., Collins P.W., Docter S., Graneto M.J., Lee L.F., Malecha J.W., Miyashiro J.M. (1997). Synthesis and biological evaluation of the 1,5-diarylpyrazole class of cyclooxygenase-2 inhibitors: Identification of 4-[5-(4-methylphenyl)-3-(trifluoromethyl)-1H-pyrazol-1-yl]benze nesulfonamide (SC-58635, celecoxib). J. Med. Chem..

[38] Kashfi K., Borgo S., Williams J.L., Chen J., Gao J., Glekas A., Benedini F., del Soldato P., Rigas B. (2005). Positional isomerism markedly affects the growth inhibition of colon cancer cells by nitric oxide-donating aspirin *in vitro* and *in vivo*. J. Pharmacol. Exp. Ther..

[39] Natarajan K., Singh S., Burke T.R., Grunberger D., Aggarwal B.B. (1996). Caffeic acid phenethyl ester is a potent and specific inhibitor of activation of nuclear transcription factor NF-kappa B. Proc. Natl. Acad. Sci. USA.

[40] Sundaresan M., Yu Z.X., Ferrans V.J., Irani K., Finkel T. (1995). Requirement for generation of H_2_O_2_ for platelet-derived growth factor signal transduction. Science.

[41] Robinson K.M., Janes M.S., Pehar M., Monette J.S., Ross M.F., Hagen T.M., Murphy M.P., Beckman J.S. (2006). Selective fluorescent imaging of superoxide *in vivo* using ethidium-based probes. Proc. Natl. Acad. Sci. USA.

